# Radiomics and Machine Learning with Multiparametric Breast MRI for Improved Diagnostic Accuracy in Breast Cancer Diagnosis

**DOI:** 10.3390/diagnostics11060919

**Published:** 2021-05-21

**Authors:** Isaac Daimiel Naranjo, Peter Gibbs, Jeffrey S. Reiner, Roberto Lo Gullo, Caleb Sooknanan, Sunitha B. Thakur, Maxine S. Jochelson, Varadan Sevilimedu, Elizabeth A. Morris, Pascal A. T. Baltzer, Thomas H. Helbich, Katja Pinker

**Affiliations:** 1 Department of Radiology, Breast Imaging Service, Memorial Sloan Kettering Cancer Center, New York, NY 10065, USA; reinerj@mskcc.org (J.S.R.); logullor@mskcc.org (R.L.G.); thakurs@mskcc.org (S.B.T.); jochelsm@mskcc.org (M.S.J.); eamorris@ucdavis.edu (E.A.M.); pinkerdk@mskcc.org (K.P.); 2 Department of Radiology, Breast Imaging Service, Guy’s and St. Thomas’ NHS Trust, Great Maze Pond, London SE1 9RT, UK; 3 Memorial Sloan Kettering Cancer Center, Sloan Kettering Institute, New York, NY 10065, USA; caleb.sooknanan@gmail.com; 4 Department of Medical Physics, Memorial Sloan Kettering Cancer Center, New York, NY 10065, USA; 5 Department of Epidemiology and Biostatistics, Memorial Sloan Kettering Cancer Center, 1275 York Ave, New York, NY 10065, USA; SevilimS@mskcc.org; 6 Department of Biomedical Imaging and Image-guided Therapy, Division of Molecular and Structural Preclinical Imaging, Medical University of Vienna, Wien 1090, Austria; pascal.baltzer@meduniwien.ac.at (P.A.T.B.); thomas.helbich@meduniwien.ac.at (T.H.H.)

**Keywords:** magnetic resonance imaging, breast cancer, radiomics, machine learning, dynamic contrast-enhanced MRI, diffusion-weighted imaging

## Abstract

The purpose of this multicenter retrospective study was to evaluate radiomics analysis coupled with machine learning (ML) of dynamic contrast-enhanced (DCE) and diffusion-weighted imaging (DWI) radiomics models separately and combined as multiparametric MRI for improved breast cancer detection. Consecutive patients (Memorial Sloan Kettering Cancer Center, January 2018–March 2020; Medical University Vienna, from January 2011–August 2014) with a suspicious enhancing breast tumor on breast MRI categorized as BI-RADS 4 and who subsequently underwent image-guided biopsy were included. In 93 patients (mean age: 49 years ± 12 years; 100% women), there were 104 lesions (mean size: 22.8 mm; range: 7–99 mm), 46 malignant and 58 benign. Radiomics features were calculated. Subsequently, the five most significant features were fitted into multivariable modeling to produce a robust ML model for discriminating between benign and malignant lesions. A medium Gaussian support vector machine (SVM) model with five-fold cross validation was developed for each modality. A model based on DWI-extracted features achieved an AUC of 0.79 (95% CI: 0.70–0.88), whereas a model based on DCE-extracted features yielded an AUC of 0.83 (95% CI: 0.75–0.91). A multiparametric radiomics model combining DCE- and DWI-extracted features showed the best AUC (0.85; 95% CI: 0.77–0.92) and diagnostic accuracy (81.7%; 95% CI: 73.0–88.6). In conclusion, radiomics analysis coupled with ML of multiparametric MRI allows an improved evaluation of suspicious enhancing breast tumors recommended for biopsy on clinical breast MRI, facilitating accurate breast cancer diagnosis while reducing unnecessary benign breast biopsies.

## 1. Introduction

Dynamic contrast-enhanced magnetic resonance imaging MRI (DCE-MRI) is the most sensitive imaging modality for breast cancer detection, outperforming mammography and ultrasound [1,2]. DCE-MRI has numerous indications in breast imaging and is widely used. DCE-MRI has excellent sensitivity, being the best available test for breast cancer detection (81–100%) [3], as well as good specificity, with pooled values around 70% [1,2,3,4,5,6,7,8,9]. Yet there are overlapping characteristics between benign and malignant abnormalities in small lesions and among high-risk women, leading to unnecessary biopsies and causing patient anxiety [10,11,12].

Diffusion-weighted imaging (DWI) has emerged as a useful technique to compensate for the lack of DCE-MRI specificity [13,14,15,16,17,18,19,20]. Thus, more and more breast MRI protocols are currently including DWI to increase specificity and reduce unnecessary benign breast biopsies [13,15,16,17,21,22,23,24,25]. Radiomics and machine learning (ML) have recently gained momentum to aid in the differentiation of breast lesions. Radiomics is an approach pertaining to the extraction and correlation of multiple imaging features which are occult to the human eye with different variables of interest (patient characteristics as well as histopathologic, genomic, molecular, or outcome data). Radiomics is typically coupled with ML methods (e.g., decision trees, support vector machines, random forests, neural networks) to select features and construct decision support models. Such models can be used for multiple purposes, including the identification of imaging characteristics that are indicative of the presence of malignancy [26,27,28,29,30,31].

To date, this strategy has mainly evaluated features extracted from DCE-MR images [32]. Only a scarce number of studies have reported the use of non-contrast sequences (e.g., DWI) in the breast to extract radiomic signatures. These have been mainly focused on the assessment of breast cancer molecular subtypes [33], lymph node metastasis prediction [34], and neoadjuvant chemotherapy response [35]. So far, data regarding the diagnostic value of multiparametric MRI radiomics from DCE and DWI are limited [23,24,28,36,37] and have not specifically focused on the challenging cases where image-guided biopsy has been recommended based on standard Breast Imaging Reporting and Database System (BI-RADS) assessment. The BI-RADS 4 category comprises a wide range of probability for malignancy, from >2% to <95%, and this category accounts for most of the false-positive cases encountered on breast MRI. Therefore, additional approaches to improve the specificity of DCE-MRI in this patient cohort are essential. DWI has been shown to add specificity but the potential of DWI radiomics and ML in this context has not been fully explored.

We hypothesized that radiomics analysis coupled with ML of multiparametric MRI may allow an improved evaluation of suspicious enhancing breast tumors recommended for biopsy on clinical breast MRI reducing the number of unnecessary biopsies. Therefore, the aim of this multicentric study was to evaluate the diagnostic accuracy of DCE and DWI radiomics models separately and combined as multiparametric MRI for the differentiation of benign and malignant tumors.

## 2. Materials and Methods

### 2.1. Study Sample

This retrospective multicenter Health Insurance Portability and Accountability Act (HIPAA)-compliant study was approved by the respective Institutional Review Boards, and the need for written informed consent was waived. Some subjects (*n* = 58) were previously reported in a different context [13].

A review of databases from Center 1, Memorial Sloan Kettering Cancer Center, spanning the period from January 2018 to March 2020, and Center 2, the Medical University of Vienna, spanning the period from January 2011 to August 2014, was performed to identify consecutive eligible patients under the following inclusion criteria: Patients older than 18 years with suspicious enhancing lesion on breast MRI categorized as Breast Imaging Reporting and Database System (BI-RADS) 4 on clinical reads who subsequently underwent image-guided biopsy of the finding. If no correlate for the suspicious enhancing mass could be identified on second-look ultrasound, the lesion was biopsied under MRI guidance. If there was a correlate on ultrasound, the lesion was biopsied under ultrasound guidance.

We excluded patients for whom examinations had no DW images or with poor image quality and breast implants. Altogether, 116 patients (mean age: 49.2 years; range: 21–89 years) with 127 lesions (mean size: 21.2 mm; range: 4–99 mm) were found eligible for this study.

### 2.2. Breast MRI Technique

In this study, 2 different scanners were used. The examinations at Center 1 were performed on 3 T MRI scanners (GE Discovery 750, GE, Milwaukee, WI, USA) using 8-channel (13 examinations, 15%) or 16-channel breast coils (Sentinelle coils, Hologic, Marlborough, MA, USA) (20 examinations, 22%) and included fat-suppressed T2-weighted fast spin echo imaging and fat-suppressed 3D T1-weighted imaging using differential subsampling with Cartesian ordering (DISCO) before and after contrast agent injection (0.1 mmol gadobutrol/kg body weight). DW images were acquired using 2 encoding schemes: Single-shot echo-planar with parallel imaging array spatial sensitivity encoding technique (ASSET) (22%) and multi-shot-multiplexed sensitivity encoding (15%). The examinations at Center 2 were acquired on a 3 T MRI scanner (Tim Trio, Siemens, Erlangen, Germany) using 4-channel breast coils (InVivo, Orlando, FL, USA) (60 examinations, 63%) and included fat-suppressed T2-weighted turbo spin echo imaging, fat-suppressed DCE T1-weighted imaging before and after contrast injection (0.1 mmol gadoterate meglumine/kg body weight), and readout-segmented echo planar imaging DWI.

In all examinations, DW images were acquired before injection of the contrast agent. Apparent diffusion coefficient (ADC) mapping was generated using built-in software. The MRI acquisition parameters for both scanners are summarized in Supplemental Tables S1 and S2.

### 2.3. Imaging Processing

Digital Imaging and Communications in Medicine (DICOM) images from early post contrast-enhanced T1-weighted imaging and DWI including ADC maps were reviewed in consensus by 2 breast radiologists (IDN and JSR), with 5 and 6 years of experience in breast imaging, respectively, using the OsiriX viewer v.9.0 (OsiriX, Geneva, Switzerland) to match lesions appropriately on the 3 sets of images. Subsequently, the same radiologists identified the lesions and performed one 3D segmentation on each set of DCE and DW images using the online available tool ITK-SNAP v3.6.0 (ITK-SNAP, Philadelphia, PA, USA). Segmentations were performed manually by delineating the borders of each lesion in every slice where it was visible to obtain a volume of interest (VOI). Equivocal cases were reviewed in consensus. In the case of DW images, VOIs were extrapolated directly to ADC maps and manually corrected in case of mismatched areas for feature extraction.

### 2.4. Radiomics Image Analysis

**Figure 1:**
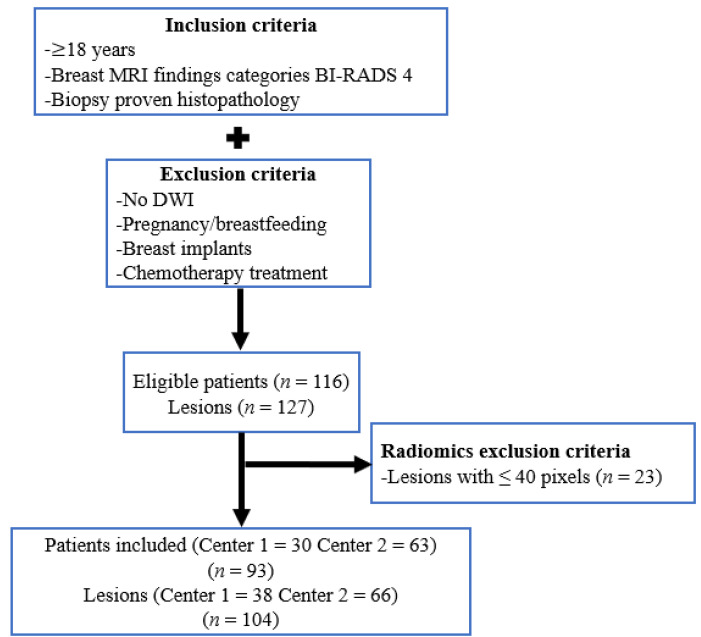
Flowchart for the study. BI-RADS, breast Imaging-Reporting and Data System; DWI, diffusion-weighted imaging.

In-house MATLAB (MathWorks Inc., Natick, MA, USA) code was used to input the VOIs extracted from DCE and DW images into the publicly available CERR (Computational Environment for Radiological Research) software (Github, San Francisco, CA, USA), which was used to calculate radiomics features [38]. Data were reduced to 16 gray levels secondary to low pixel count in some lesions, and only an interpixel distance of 1 was considered, ensuring reasonable counting statistics for texture feature calculation. To improve the prediction model, only lesions with more than 40 pixels were considered, resulting in the exclusion of 23 patients with 23 lesions. Eventually, 93 patients (30 from Center 1 and 63 from Center 2) with 104 lesions (38 from Center 1 and 66 from Center 2) were included for the analysis. In total, 11 patients showed more than 1 lesion on MRI. Figure 1 shows a flowchart with the selection of patients for the study.

Radiomic features were calculated using the gray level co-occurrence matrix (GLCM), gray level run length matrix (RLM), gray level size zone matrix (SZM), neighborhood gray level dependence matrix, neighborhood gray tone difference matrix, and first-order statistics.

### 2.5. Reference Standard

The reference standard was histopathology-established by image-guided biopsy. In patients whose biopsy yielded a benign high-risk lesion (e.g., intraductal papilloma, atypical ductal hyperplasia, or atypical lobular hyperplasia), the histological report from the surgical biopsy was reviewed to confirm the benignity.

### 2.6. Statistical Analysis and Predictive Model Building

Continuous variables were summarized using means (±SD) and medians (range), and categorical variables were summarized using proportions.

To account for possible site variations, radiomics features underwent Combat harmonization prior to subsequent analysis [39].

For radiomics analysis, statistical analysis was performed using SPSS (version 25, IBM Corp., Armonk, NY, USA). Univariable analysis was performed to identify radiomic features that were significantly different between malignant and benign lesions. Since the number of patients in each group was not large (especially after imposing lesion size restrictions), the Mann–Whitney U-test for 2 independent samples was used to determine significant differences in all lesions. *p* values < 0.05 were considered significant. Supplemental Tables S3 and S4 show univariable *p*-values for DWI and DCE radiomics features, and univariable correlation analysis between radiomic features calculated from DWI and DCE data respectively.

To proceed with multivariable analysis, model overfitting was prevented by reducing the number of parameters through feature selection using cross-validated least absolute shrinkage and selection operator (LASSO) regression. Only the top 5 parameters were selected for model development to ensure sufficient lesions per parameter for the minority class. Significant radiomic features were then incorporated into multivariable modeling to produce a robust ML model for discriminating between benign and malignant lesions. Z-score normalization of selected features was utilized in model development to account for the various degrees of magnitude encountered in radiomics. A medium Gaussian support vector machine (SVM) model with 5-fold cross validation was employed to develop the predictive models derived from DCE and DW images as well as a combination of both datasets. This process was performed 1000 times for each set of features (DWI, DCE, combined) to determine aggregate diagnostic metrics. The area under the receiver operating characteristic curve (AUC) was used to evaluate the performance of the models and was compared for all 3 models [40]. Sensitivity, specificity, positive predictive value (PPV), negative predictive value (NPV), and accuracy were calculated. *p* values < 0.05 were considered significant.

AUC values were statistically compared utilizing the methodology devised by Hanley and McNeil [41], which was specifically designed to evaluate ROC curves derived from the same cases.

## 3. Results

### 3.1. Patient Sample and Breast Lesion Characteristics

The final patient sample consisted of 93 patients (mean age: 48.5 years ± 12 years; 93 women) with 104 lesions (mean size: 22.8 mm; range: 7–99 mm). These lesions comprised 46 malignant lesions (mean size: 28.8 mm; range: 10–99 mm), of which 35 were masses and 11 non-mass lesions (NMLE). Lesions with benign histopathology numbered 58 (mean size: 18.2 mm; range: 7–51 mm), of which 50 were masses and the remaining 8 were NMLE. This number represented clinically a false-positive rate of 55.7% in this cohort. Characteristics for patients and lesions included in the analysis are summarized in Table 1 and Table 2.

### 3.2. Radiomics Analysis for Breast Lesion Differentiation

After segmentation, the median benign lesion size was 255 pixels (range: 40–5379 pixels) and the median malignant lesion size was 2104 pixels (range: 115–58,485 pixels).

CERR analysis resulted in 102 radiomic features subdivided into 6 categories: 22 based on first-order statistics, 26 based on GLCM, 16 based on RLM, 16 based on SZM, 17 based on neighborhood gray level dependence matrix, and 5 based on neighborhood gray tone difference matrix.

**Figure 2:**
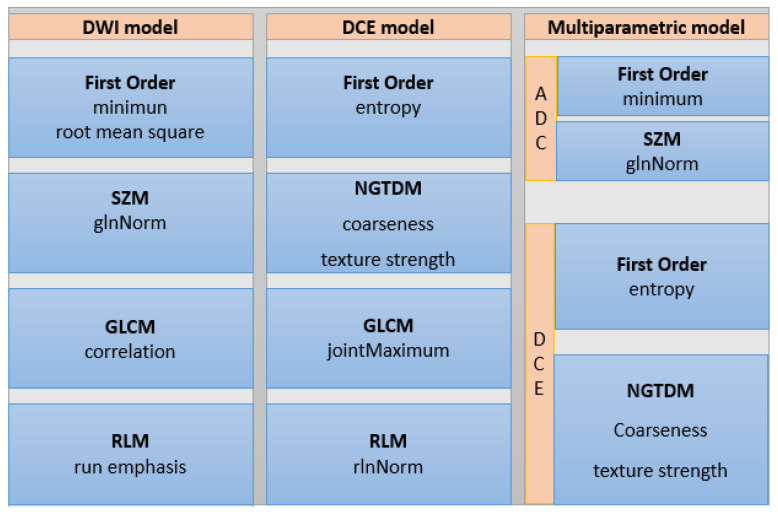
Radiomic features of interest per class and dataset after least absolute shrinkage and selection operator (LASSO) regression. GLCM, gray level co-occurrence matrix; RLM, run length matrix; SZM, size zone matrix; NGDTM, neighborhood gray tone difference matrix; DWI, diffusion-weighted imaging; DCE, dynamic contrast-enhanced; glnNorm, gray level non-uniformity normalized; rlnNorm, run length non-uniformity normalized.

**Figure 3:**
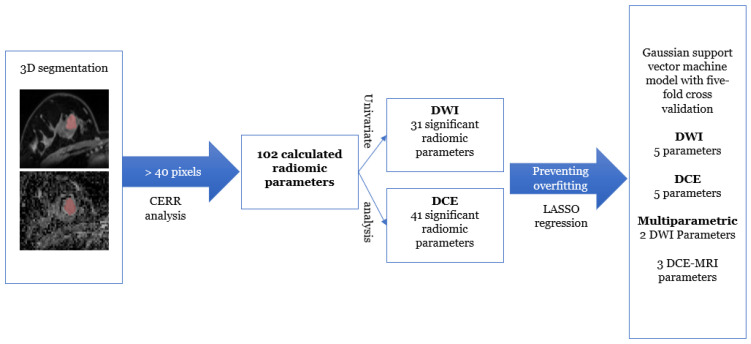
Workflow for the radiomics analysis. DCE-MRI, dynamic contrast-enhanced MRI; DWI, diffusion-weighted imaging; CERR, Computational Environment for Radiological Research; LASSO, least absolute shrinkage and selection operator.

At univariable analysis, 34 and 27 radiomic features were found to be significantly different between benign and malignant lesions on the DWI and DCE datasets, respectively. With this number of significant features, LASSO regression was applied, reducing the number of features of interest to five (from five classes) for subsequent multivariable modeling. Figure 2 shows the radiomic features of interest per class and the dataset after LASSO regression. Using a medium Gaussian SVM model with five-fold cross validation with all five parameters for DWI, DCE, and multiparametric datasets, the following diagnostic metrics were achieved for the separation of benign and malignant lesions. Figure 3 shows the workflow chart for the radiomics analysis, and the metrics for the performance of the models for each dataset are summarized in Table 3.

When using DCE data, a sensitivity of 76.6% (95% CI: 62.0–87.7), specificity of 77.2% (95% CI: 64.2–87.3), diagnostic accuracy of 76.9% (95% CI: 67.6–84.6), PPV of 73.5% (95% CI: 62.6–82.1), NPV of 80.0% (95% CI: 70.1–87.2), and AUC of 0.83 (95% CI: 0.75–0.91) were obtained for the developed model. There were 11 false-negative (FN) lesions and 13 false-positive (FP) lesions. The mean size of FN lesions was 20.6 mm (range: 9–34 mm) with three NMLEs. The mean size of FP lesions was 23.4 mm (range: 11–51 mm) with two NMLEs.

The model utilizing the ADC maps derived from DWI dataset achieved a sensitivity of 68.1% (95% CI: 52.8–80.9), specificity of 77.2% (95% CI: 64.2–87.3), diagnostic accuracy of 73.1% (95% CI: 63.5–81.3), PPV of 71.1% (95% CI: 59.5–80.5), NPV of 74.6% (95% CI: 65.4–82.0), and AUC of 0.79 (95% CI: 0.70–0.88). This model misclassified 28 lesions, accounting for 15 FN lesions and 13 FP lesions. The mean size of FN lesions was 22.2 mm (range: 15–42 mm) with one NMLE. The mean size of FP lesions was 17.8 mm (range: 9–51 mm) with four NMLEs.

The multiparametric dataset tended to show higher values, with a sensitivity of 85.1% (95% CI: 71.7–93.8), specificity of 79.0% (95% CI: 66.1–88.6), diagnostic accuracy of 81.7% (95% CI: 73.0–88.), PPV of 76.9% (95% CI: 66.5–84.8), NPV of 86.5% (95% CI: 76.2–92.8), and AUC of 0.85 (95% CI: 0.77–0.92). This model classified 81.7% of the lesions correctly, yielding the lowest number of FN lesions (*n* = 7) and FP lesions (*n* = 12). The mean size of FN lesions was 20.7 mm (range: 9–34 mm), and the mean size of FP lesions was 24.9 mm (range: 11–51 mm). There were all masses except for two NMLEs in each group.

**Figure 4:**
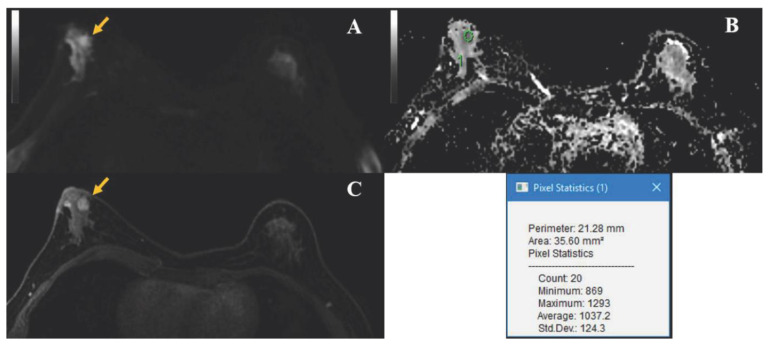
Axial MR images of a 40-year-old woman with a 9 mm benign mass, of which the biopsy yielded intraductal papilloma (yellow arrows). (**A**) Axial diffusion-weighted imaging (DWI) at a b value of 800 s/mm^2^ shows a hyperintense lesion in the anterior third of the right breast. (**B**) Correlative parametric apparent diffusion coefficient (ADC) map with a region of interest (ROI) placed within the lesion and ROI information. ADC values are expressed in mm^2^/s. (**C**) Axial Dynamic contrast-enhanced image depicts a slightly irregular, heterogeneous enhancing mass corresponding to the hyperintense lesion on DWI. This lesion had suspicious characteristics on dynamic contrast-enhanced images and low ADC values and was therefore categorized as BI-RADS 4 in a clinical read, subsequently undergoing a biopsy. Multiparametric radiomics accurately classified the lesion as benign.

Figure 4 shows a lesion that, on clinical qualitative assessment by the radiologists’ review, was incorrectly classified as suspicious. On subsequent biopsy, the lesion yielded benign pathology. Multiparametric radiomics accurately classified the lesion as benign.

Comparison of AUC values for all three models showed no significant differences in DWI data compared with DCE data (DWI 0.79 ± 0.05, DCE 0.83 ± 0.04, *p* = 0.48), DWI data compared with combined data (DWI 0.79 ± 0.05, combined 0.85 ± 0.04, *p* = 0.16), and DCE data compared with combined data (DCE 0.83 ± 0.04, combined 0.85 ± 0.04, *p* = 0.70).

## 4. Discussion

In this study, we investigated the diagnostic value of radiomics analysis when coupled with ML of DCE, ADC maps derived from DWI, and combined as multiparametric MRI for the evaluation of suspicious enhancing breast tumors recommended for biopsy on clinical breast MRI. When coupled with ML with multiparametric MRI, radiomics tended to improve breast cancer diagnosis, maximizing the accuracy for the differentiation of benign and malignant tumors. Our results also showed that it has the potential to serve as a decision-supporting tool to reduce unnecessary biopsies in benign breast tumors.

We developed a multiparametric radiomics model which, despite achieving the best AUC (0.85), compared to models based solely on features extracted from DCE and DWI, it did not achieve statistically significant difference. However, this multiparametric model allowed for the best diagnostic accuracy (81.7%), helping in the correct classification of suspicious enhancing breast tumors recommended for biopsy over clinical qualitative assessment by the radiologists’ review. The developed model reduced the number of FP lesions and thus would have allowed a reduction of unnecessary benign breast biopsies.

The diagnostic model based on DCE data achieved an accuracy of 76.9% and AUC of 0.83. This model misclassified 24 lesions, yielding 13 FP lesions. In a previous study [42], Nie et al. used an artificial neural network for diagnostic feature selection of quantitative morphology and texture features of breast lesions, reporting a similar AUC of 0.86 for lesion differentiation. Notably, they included histologically proven benign and malignant lesions, but those presenting as diffuse infiltrating enhancements or ill-defined tumor margin were excluded. These most often represent the diagnostically challenging tumors and were included in our patient collective. Truhn et al. [43] compared the diagnostic performance of radiomic analysis and a convolutional neural network (CNN) model to three breast radiologists for the classification of enhancing lesions in clinical MRI. They reported that the performance of the CNN model was superior to radiomics analysis with an AUC of 0.88, but the CNN model did not outperform breast radiologist interpretation of multiparametric MRI with an AUC of 0.98. Truhn et al. included the whole spectrum of lesions ranging from benign, probably benign, suspicious, and highly suggestive of malignancy to biopsy-proven cancers (BI-RADS 2 to 6), whereas our focused on suspicious lesions only, which explains their superior performance.

Gibbs et al. [44] evaluated the utility of radiomics analysis for breast cancer diagnosis in small breast lesions (BI-RADS 4/5) using radiomics DCE-based parameter maps and achieved an AUC of 0.78. Lo Gullo et al. [45] focused on the characterization of subcentimeter breast masses (BI-RADS 3/4) in high-risk patients. Radiomics analysis coupled with machine learning showed a diagnostic accuracy of 81.5%, improving lesion characterization compared with radiologists’ BI-RADS classification. Our results for the models for DCE alone are in good agreement with these studies, but none of these studies have included DWI.

The diagnostic model utilizing DWI data achieved a diagnostic accuracy of 73.1% and AUC of 0.79. This model misclassified more lesions than the one based solely on DCE data, yielding 13 FP lesions. This could be explained by a lower resolution of DWI compared with DCE-MRI, which has a smaller number of pixels segmented per lesion. In our study, ADC maps were used to build the DWI model since it is believed that they yield more accurate results. High b-value DW images are susceptible to the T2 shine-through effect and may not reflect truly solid tumor components or areas with hindered diffusivity [33].

Bickelhaupt et al. used kurtosis DWI on MRI to generate a radiomic model to help clarify findings suspicious for cancer in mammography [23]. This model reduced the number of FP lesions with an improved specificity (70%) compared to median ADC and apparent kurtosis coefficient alone. It must be noted that this study focused on lesions that were suspicious on mammography. In this setting, MRI can often either solidify, elevate, or lower the level of suspicion. In our study, the clinical scenario was different as we focused on MRI BI-RADS 4 lesions, which are often occult on mammography and sonography. Thus none of these modalities can be incorporated into the decision-making process.

The model based on multiparametric MRI tended to show the best diagnostic accuracy (81.7%) and AUC (0.85). Parekh et al. [36] evaluated the diagnostic capabilities of radiomic feature maps derived from radiomics analysis of ADC maps and DCE-MRI with pharmacokinetic modeling. They demonstrated differences in radiomic feature map curves for benign and malignant lesions, with an increased entropy in malignant tumors. Their model, which included quantitative MRI metrics of ADC and perfusion, achieved an AUC of 0.91 with a sensitivity of 93% and specificity of 85%. Zhang et al. [46] investigated T2-weighted imaging, T1-weighted imaging, quantitative pharmacokinetic parameters of DCE-MRI, and diffusion kurtosis imaging (DKI) with ADC mapping to build models for the differentiation of breast lesions based on each sequence or combinations of sequences. The optimal radiomics model included T2-weighted imaging, DKI, and quantitative DCE-MRI parameter maps, yielding an AUC of 0.921 with an accuracy of 0.833. These previous studies have again included BI-RADS 2–6 lesions as opposed to suspicious lesions only, which can explain the better classification accuracies achieved.

Verburg et al. used computer-aided diagnosis (CAD) from multiparametric MRI (T2-weighted, DWI, T1-weighted DCE at high spatial and at high temporal resolution) to predict which BI-RADS 3 or 4 lesions are benign [47]. Whereas the aim in this study was different—to identify lesions that could be prevented from being recalled in the screening of women with extremely dense breasts (DENSE Trial)—the results are similar to our own in the sense that dedicated radiomics and ML has the potential to reduce false-positive diagnoses and, consequently, to reduce the number of biopsies.

The multiparametric radiomic model reduced the number of FP lesions to 12, while in clinical practice, radiologists incorrectly classified 58 benign lesions as FP lesions, which subsequently underwent unnecessary biopsies. Although the multiparametric model reduced the number of FN lesions, it misclassified seven cancers, which included two NMLEs. We suggest that the added value of the current multiparametric model could be as an adjunct decision-supporting tool for lesions of lower clinical suspicion to decide on follow-up rather than biopsy.

It is worth highlighting that our study included data from different MRI protocols and scanners across two different institutions. This could potentially introduce weakness, e.g., data noise or dilution of the association by the protocol/image quality differences, but has a positive impact in the generalizability of the results.

Nevertheless, there are limitations. Our study sample included subcentimeter benign breast lesions with a median pixel size lower than for cancers, which do not contribute many pixels to the final VOIs. This can lead to an increased proportion of pixels that can be regarded as potentially contaminated by partial volume effects. To tackle this limitation and ensure adequate counting statistics, we lowered the data to only 16 gray levels (vs 32 or 64 gray levels which have previously been employed in breast MRI) and included only lesions with more than 40 pixels. VOIs from breast lesions were obtained manually. Our strict inclusion criteria afforded a relatively small sample size of 104 breast lesions. This small sample size precluded separation of data into training and test sets. Thus, the developed models require further validation in larger multicenter studies.

## 5. Conclusions

In conclusion, radiomics analysis coupled with machine learning of multiparametric MRI allows an improved evaluation of suspicious enhancing breast tumors recommended for biopsy on clinical breast MRI and has the potential to serve as a decision-supporting tool to reduce unnecessary biopsies in benign breast tumors.

## Figures and Tables

**Table 1:** Characteristics of the 93 patients included in the analysis.

Patient Characteristic	Number (Percentage)
Mean age (years; SD)	49 years ± 12 years
Menopausal status	
Fertile	55 (59.1%)
Menopause	38 (40.9%)
Breast Findings	
Benign	58 (55.8%)
Malignant	46 (44.2%)

**Table 2:** Characteristics of the 104 lesions included in the analysis.

Benign Lesions			Malignant Lesions		
Mass	50 (86.2%)		Mass		35 (76%)
NMLE	8 (13.8%)		NMLE		11(24%)
Histopathology					
Fibroadenoma or fibroadenomatoid change		30 (51.8%)	IDC	Histological Grade 1: 4 (8.6%)	
Phyllodes tumor		1 (1.7%)		Histological Grade 2: 18 (39.2%)	
Adenosis, stromal fibrosis, ductal ectasia, or normal breast parenchyma		10 (17.3%)		Histological Grade 3: 20 (43.6%)	
FCC		5 (8.6%)	ILC	2 (4.3%)	
ADH or ALH		4 (6.9%)			
PASH		3 (5.2%)			
Papilloma		2 (3.4%)			
Hamartoma		1 (1.7%)	DCIS	2 (4.3%)	
Fat necrosis		1 (1.7%)			
Epithelial intraductal proliferation without atypia		1 (1.7%)			

NMLE, non-mass lesion; FCC, fibrocystic changes; ADH, atypical ductal hyperplasia; ALH, atypical lobular hyperplasia; PASH, pseudoangiomatous stromal hyperplasia; IDC, invasive ductal carcinoma; ILC, invasive lobular carcinoma; DCIS, ductal carcinoma in situ.

**Table 3:** Diagnostic metrics for the performance of the machine learning (ML) models for each dataset.

Dataset	Sensitivity	Specificity	PPV	NPV	Accuracy	AUC
	(95% CI)	(95% CI)	(95% CI)	(95% CI)	(95% CI)	(95% CI)
DWI	68.1 (52.8–80.9)	77.2 (64.2–87.3)	71.1 (59.5–80.5)	74.6 (65.4–82.0)	73.1 (63.5–81.3)	0.79 (0.70–0.88)
DCE	76.6 (62.0–87.7)	77.2 (64.2–87.3)	73.5 (62.6–82.1)	80 (70.1–87.2)	76.9 (67.6–84.6)	0.83 (0.75–0.91)
Multiparametric	85.1 (71.7–93.8)	79 (66.1–88.6)	76.9 (66.5–84.8)	86.5 (76.2–92.8)	81.7 (73.0–88.6)	0.85 (0.7–0.92)

Abbreviations: DWI, diffusion-weighted imaging; DCE, dynamic contrast-enhanced; CI, confidence interval; PPV, positive predictive value; NPV, negative predictive value; AUC, area under the curve.

## Data Availability

The data presented in this study are available upon reasonable request from the corresponding author.

## Supplementary Materials

The following are available online at https://www.mdpi.com/article/10.3390/diagnostics11060919/s1, Table S1: Summary of imaging protocols and acquisition parameters, Table S2: Summary of DWI protocols and acquisition parameters, Table S3: Univariable *p*-values for DWI and DCE radiomics features, Table S4: Univariable correlation analysis between radiomic features calculated from DWI and DCE data.
