# Supplementary material for: Radiomics and Machine Learning with Multiparametric Breast MRI for Improved Diagnostic Accuracy in Breast Cancer Diagnosis
Source: Diagnostics (Basel). 2021 May 21;11(6):919. doi: 10.3390/diagnostics11060919 (PMC8223779; doi:10.3390/diagnostics11060919)
Supplement: Supplementary file 1 [file diagnostics-11-00919-s001.zip › diagnostics-1201978-supplementary.pdf]

Table S1. Summary of imaging protocols and acquisition parameters

Scanner	3 T MRI Tim Trio, Siemens			3 T GE Discovery 750, GE		
Sequence	T2-weighted turbo spin echo	T1-weighted VIBE	T1-weighted turbo FLASH-3D	T2-weighted fast spin echo	T1-weighted	T1-weighted DISCO
Fat suppression	Nonselective inversion	Frequency selective	Frequency selective	Inversion recovery	Inversion recovery	Inversion recovery
Repetition time (msec)	4800	3.61	877	6460	7.9	7.9
Echo time (msec)	61	1.4	3.82	104.1	4.3	4.3
Matrix size (mm)	512 × 512	512 × 512	512 × 512	512 × 512	512 × 512	512 × 512
Resolution (mm)	1 × 1 × 4	1.7 × 1.7 × 1.7	1 × 1 × 1	1 × 1 × 3	1 × 1 × 1	1 × 1 × 1
Parallel imaging	GRAPPA 2	GRAPPA 2	GRAPPA 2	ASSET	ASSET	ASSET
Image acquisition time (min:sec)	2:26	0:13	2:00	2:32	1:30	4:30
Number of lesions	65			39		

Abbreviations: ASSET, array spatial sensitivity encoding technique; VIBE, Volumetric interpolated breath-hold examination; FLASH, fast low-angle shot; DISCO, Differential Sub-sampling with Cartesian Ordering.

Table S2. Summary of DWI protocols and acquisition parameters

Scanner	3 T Tim Trio, Siemens	3 T GE Discovery 750, GE	
Sequence	Axial readout segmented echo-planar imaging	Axial single-shot DWI ASSET echo planar imaging	Axial multiplexed sensitivity-encoding DWI
Diffusion directions	Three-direction trace	Three-direction trace	Three-direction trace
b value (s/mm2)	0, 850	0, 800	0, 800
Fat suppression	Inversion recovery; gradient reversal	Inversion recovery	Inversion recovery
Repetition time (msec)	8000	6000	2000-17000
Echo time (msec)	Minimum	Minimum	Minimum
Inversion time (msec)	210	210	210
Field of view (mm)	360 × 202	340 × 320	340 × 320
Matrix	172 × 96	256 × 256	300 × 300
Section thickness (mm)	5	3.9	3.9
Intersection gap (mm)	5	3.9	3.9
N° of readout segments	5	1	4
N° of sections	24	34	34

Phase-encoding direction	anteroposterior	anteroposterior	anteroposterior
Time of scan (min:s)	2:56	4:02	6:04
Number of lesions	65	21	18

Abbreviations: ASSET, array spatial sensitivity encoding technique; DWI, diffusion-weighted imaging.

Table S3. Univariable p-values from Mann-Whitney analysis differentiating benign from malignant lesions for all calculated radiomics features determined utilizing MRI acquired DWI and DCE data.

Radiomics Feature	DWI (*p*-value)	DCE (*p*-value)
First Order		
minimum	0.003	0.466
maximum	0.827	0.188
mean	0.005	0.257
range	0.034	0.197
standard deviation	0.082	0.268
variance	0.105	0.541
median	0.009	0.231
skewness	0.007	0.039
kurtosis	0.260	0.001
entropy	0.011	0.003
root mean square	0.002	0.252
energy	0.398	0.524
total energy	0.650	0.257
mean absolute deviation	0.045	0.416
median absolute deviation	0.036	0.427
10th percentile	0.011	0.390
90th percentile	0.015	0.244
robust mean absolute deviation	0.052	0.899
robust median absolute deviation	0.042	0.888
interquartile range	0.049	0.971
coefficient of dispersion	0.014	0.491
coefficient of #variation	0.027	0.935
Gray level cooccurrence matrix		
energy	0.951	0.002
joint entropy	0.015	0.001
joint maximum	0.678	0.013
joint average	0.014	0.154
joint variance	0.221	0.010
contrast	0.935	0.077
inverse difference moment	0.868	0.127
inverse difference moment normalized	0.914	0.091
inverse difference	0.971	0.131
inverse difference normalized	0.585	0.099
inverse variance	0.483	0.017
dissimilarity	0.868	0.077
difference entropy	0.546	0.047
difference variance	0.604	0.084
difference average	0.868	0.077
sum average	0.014	0.154

sum variance	0.143	0.023
sum entropy	0.047	0.003
correlation	0.033	0.581
cluster tendency	0.143	0.023
cluster shade	0.173	0.546
cluster prominence	0.073	0.112
Haralick correlation	0.904	0.089
auto correlation	0.015	0.155
first information measure of correlation	0.030	0.073
second information measure of correlation	0.280	0.310
Run length matrix		
gray level nonuniformity	0.046	<0.0005
gray level nonuniformity normalized	0.099	0.004
gray level variance	0.268	0.175
high gray level run emphasis	0.004	0.106
low gray level run emphasis	0.265	0.401
long run emphasis	0.817	0.807
long run high gray level emphasis	0.016	0.383
long run low gray level emphasis	0.310	0.143
run emphasis	0.001	0.260
run length nonuniformity	0.012	<0.0005
run length nonuniformity normalized	0.904	0.173
run length variance	0.919	0.722
run percentage	0.857	0.390
short run emphasis	0.888	0.169
short run high gray level emphasis	0.008	0.071
short run low gray level emphasis	0.474	0.516
Size zone matrix		
small zone emphasis	0.157	0.707
large zone emphasis	0.359	0.053
gray level nonuniformity	0.018	<0.0005
gray level nonuniformity normalized	<0.0005	0.086
size zone nonuniformity	0.001	<0.0005
size zone nonuniformity normalized	0.226	0.781
zone percentage	0.961	0.455
low gray level large zone emphasis	0.756	0.524
high gray large zone emphasis	0.005	0.048
small zone large gray level emphasis	0.817	0.599
small zone high gray level emphasis	0.059	0.039
large zone low gray level emphasis	0.930	0.171
large zone high gray level emphasis	0.100	0.007
gray level variance	0.319	0.529
size zone variance	0.405	0.021
zone emphasis	0.088	0.124
Neighborhood gray level dependence matrix		
low dependence emphasis	0.807	0.458
high dependence emphasis	0.595	0.474
low gray level count emphasis	0.427	0.355
high gray level count emphasis	0.012	0.165

low dependence low gray level emphasis	0.971	0.533
low dependence high gray level emphasis	0.096	0.171
high dependence low gray level emphasis	0.313	0.076
high dependence high gray level emphasis	0.076	0.427
gray level nonuniformity	0.082	<0.0005
gray level nonuniformity normalized	0.645	0.010
dependence count nonuniformity	0.003	0.000
dependence count nonuniformity normalized	0.641	0.313
gray level variance	0.246	0.077
dependence count variance	0.366	0.503
entropy	0.077	0.058
energy	0.221	0.055
Neighborhood gray tone difference matrix		
coarseness	0.061	<0.0005
contrast	0.161	0.115
busyness	0.712	<0.0005
complexity	0.195	0.094
texture strength	0.063	<0.0005

Abbreviations: DCE, dynamic contrast-enhanced; DWI, diffusion-weighted imaging.

Table S4. Univariable correlation coefficients and corresponding *p*-values from Spearman rank analysis between radiomic features acquired utilzing DWI and DCE data.

Radiomics Feature	Correlation Coefficient	*p*-value
First Order		
minimum	0.169	0.086
maximum	-0.128	0.197
mean	-0.011	0.913
range	-0.059	0.551
standard deviation	0.004	0.970
variance	-0.056	0.573
median	0.010	0.923
skewness	0.037	0.710
kurtosis	0.187	0.057
entropy	0.016	0.869
root mean square	-0.018	0.853
energy	0.182	0.064
total energy	0.010	0.919
mean absolute deviation	0.010	0.919
median absolute deviation	-0.005	0.963
10th percentile	0.054	0.585
90th percentile	-0.028	0.777
robust mean absolute deviation	0.033	0.739
robust median absolute deviation	0.015	0.878
interquartile range	0.017	0.860
coefficient of dispersion	0.288	0.003

coefficient of #variation	0.271	0.005
Gray level cooccurrence matrix		
energy	0.085	0.392
joint entropy	0.137	0.166
joint maximum	0.091	0.360
joint average	0.036	0.715
joint variance	0.076	0.440
contrast	0.220	0.025
inverse difference moment	0.344	<0.0005
inverse difference moment normalized	0.230	0.019
inverse difference	0.341	<0.0005
inverse difference normalized	0.323	0.001
inverse variance	0.215	0.028
dissimilarity	0.304	0.002
difference entropy	0.237	0.016
difference variance	0.116	0.239
difference average	0.304	0.002
sum average	0.036	0.715
sum variance	0.075	0.450
sum entropy	0.086	0.383
correlation	0.195	0.047
cluster tendency	0.075	0.450
cluster shade	-0.050	0.612
cluster prominence	0.088	0.372
Haralick correlation	0.074	0.456
auto correlation	0.022	0.821
first information measure of correlation	-0.075	0.450
second information measure of correlation	-0.062	0.533
Run length matrix		
gray level nonuniformity	0.764	<0.0005
gray level nonuniformity normalized	0.020	0.843
gray level variance	0.038	0.699
high gray level run emphasis	0.031	0.757
low gray level run emphasis	0.017	0.865
long run emphasis	0.295	0.002
long run high gray level emphasis	0.057	0.563
long run low gray level emphasis	-0.032	0.745
run emphasis	0.419	<0.0005
run length nonuniformity	0.801	<0.0005
run length nonuniformity normalized	0.333	0.001
run length variance	0.283	0.004

run percentage	0.335	0.001
short run emphasis	0.325	0.001
short run high gray level emphasis	0.088	0.374
short run low gray level emphasis	0.032	0.746
Size zone matrix		
small zone emphasis	0.252	0.010
large zone emphasis	0.246	0.012
gray level nonuniformity	0.749	<0.0005
gray level nonuniformity normalized	-0.097	0.325
size zone nonuniformity	0.645	<0.0005
size zone nonuniformity normalized	0.281	0.004
zone percentage	0.393	<0.0005
low gray level large zone emphasis	-0.015	0.877
high gray large zone emphasis	0.032	0.750
small zone large gray level emphasis	0.021	0.833
small zone high gray level emphasis	0.076	0.444
large zone low gray level emphasis	0.048	0.631
large zone high gray level emphasis	0.346	<0.0005
gray level variance	-0.027	0.785
size zone variance	0.194	0.049
zone emphasis	0.678	<0.0005
Neighborhood gray level dependence matrix		
low dependence emphasis	0.375	<0.0005
high dependence emphasis	0.309	0.001
low gray level count emphasis	-0.010	0.922
high gray level count emphasis	0.030	0.761
low dependence low gray level emphasis	0.238	0.015
low dependence high gray level emphasis	0.302	0.002
high dependence low gray level emphasis	-0.001	0.995
high dependence high gray level emphasis	0.083	0.401
gray level nonuniformity	0.588	<0.0005
gray level nonuniformity normalized	0.095	0.338
dependence count nonuniformity	0.822	<0.0005
dependence count nonuniformity normalized	0.349	<0.0005
gray level variance	0.065	0.513
dependence count variance	0.246	0.012
entropy	0.496	<0.0005
energy	0.339	<0.0005
Neighborhood gray tone difference matrix		
coarseness	0.656	<0.0005

contrast	0.241	0.014
busyness	0.186	0.059
complexity	0.109	0.271
texture strength	0.679	<0.0005

Abbreviations: DCE, dynamic contrast-enhanced; DWI, diffusion-weighted imaging.
